# Psychiatric reaction of an intensive care unit survivor in the context of coronavirus disease 2019: a case report

**DOI:** 10.1186/s13256-022-03450-3

**Published:** 2022-06-23

**Authors:** Lamyae Benzakour, Emmanouela Kakoraiti, Alexandre Perrin, Sara Cereghetti, Frédéric Assal

**Affiliations:** 1 Department of Psychiatry, Liaison Psychiatry Unit, University Hospitals of Geneva, 4, Rue Gabrielle Perret-Gentil, 1205 Geneva, Switzerland; 2 Dpartment of Medicine, Intensive Care Service, University Hospitals of Geneva, 4, Rue Gabrielle Perret-Gentil, 1205 Geneva, Switzerland; 3 Department of Neurosciences, Service of Neurology, University Hospital of Geneva, 4, Rue Gabrielle Perret-Gentil, 1205 Geneva, Switzerland

**Keywords:** Case report, Liaison psychiatry, COVID-19, Acute stress disorder (ASD), Posttraumatic stress disorder (PTSD)

## Abstract

**Background:**

The coronavirus disease 2019 pandemic has had a serious impact on global mental health, particularly in intensive care unit survivors. Given the lethal potential and unpredictability of coronavirus disease 2019, a high risk of posttraumatic stress disorder was identified in the beginning of the crisis. There are insufficient details in current literature and no official guidelines available for the treatment and follow-up of acute stress disorder and the prevention of posttraumatic stress disorder for intensive care unit survivors in the context of coronavirus disease 2019.

**Case presentation:**

We hereby describe a 67-year-old Swiss patient presenting a psychiatric reaction in the context of coronavirus disease 2019. He was admitted to the intensive care unit due to severe acute respiratory distress syndrome from severe acute respiratory syndrome coronavirus 2 and intubated for 13 days. Afterwards, there was a severe worsening of acute renal failure prompting hemodialysis, and he developed delirium. Psychiatric liaison was requested 4 days post-intubation because the patient presented residual symptoms of delirium, false memories about the real context of his medical care, and ideas of persecution toward medical caregivers. He suffered from a very strong peritraumatic reaction, then developed an acute stress disorder linked with his care on the intensive care unit. We looked for strategies to prevent progression from acute stress disorder to posttraumatic stress disorder. We proceeded to the following therapeutic interventions: intensive psychiatric follow-up, intensive care unit diary, and low-dose antipsychotic treatment. The aim of our psychotherapeutic approach was to allow him to increase his feeling of security and to cope with the reality of his traumatic experience. He showed clinical improvement in his mental state after 3 months, despite several predictive factors of evolution to post-intensive care unit posttraumatic stress disorder.

**Conclusion:**

This case report illustrates how a delusional clinical presentation after intensive care in the context of coronavirus disease 2019 can hide psychotraumatic symptoms. It is important to highlight that the intensive care unit diary completed by the intensive care team and the follow-up by the psychiatric liaison team helped the patient reconstruct an appropriate and coherent account. Further studies are needed to determine the psychiatric effects of coronavirus disease 2019 and to assess early and appropriate psychiatric intervention for patients hospitalized for coronavirus disease 2019 to prevent posttraumatic stress disorder.

## Background

The COVID-19 pandemic represents an exceptional global crisis that raises many questions among the international medical community. Its psychiatric consequences are still being explored. Given the lethal potential and the unpredictability of COVID-19, a high risk of posttraumatic stress disorder (PTSD) was identified in the beginning of the crisis [1]. It is estimated that 5% of patients severely affected by COVID-19 were admitted to an intensive care unit (ICU) [2], and more than 80% of them developed delirium in one study [3]. Adult ICU survivors are at high risk of developing neuropsychiatric complications, such as post intensive care syndrome (PICS) at 19–22% [4]. PICS constitutes new or worsening cognitive and/or psychiatric and/or physical functions, usually identified in the period immediate following critical illness. Psychiatric manifestations of PICS include anxiety, depression, and posttraumatic stress disorder (PTSD) [5]. PTSD can occur as a consequence of acute stress disorder (ASD). The latter is characterized by acute stress reactions that may occur in the initial month after a person is exposed to a traumatic event. The disorder includes symptoms of intrusion, dissociation, negative mood, avoidance, and arousal. Early identification and treatment of severe acute stress responses can have the additional benefit of limiting subsequent PTSD, which is diagnosed after 4 weeks of symptoms following exposure to trauma. The experience of the disease itself can be a traumatic event and lead to PTSD, and several risk factors of developing PTSD after a trauma event are known [6]. Before the trauma event, peritrauma factors are history of disease, previous psychiatric disorders, and childhood traumas. During the trauma event, peritrauma factors include peritraumatic dissociation. After the trauma event, posttrauma factors are acute stress disorder, acute stress symptoms, anxiety, avoidance, and depression [6]. The risk of developing PTSD seems to be high for adults surviving COVID-19, with a rate of 28% reported in a recent study [7]. In this case report, we illustrate the direct and indirect effects of stress from COVID-19 on the mental health of a 67-year-old Swiss patient who was hospitalized in the ICU for COVID-19 and how this complex clinical situation with a high risk of developing PTSD was managed. This paper describes a complex case of acute COVID-19 in intensive care unit associated with psychiatric issues and its clinical follow-up at 3 months during the first wave. This case is rare because it shows how direct and indirect effects of SARS-CoV-2 on the brain can interplay with the traumatic load related to the COVID-19 experience and how to manage the treatment with a 3-month follow-up. To the best of the authors’ knowledge, there are no case reports to date describing acute stress disorder in a COVID-19 survivor.

## Case presentation

### Patient information

The reported patient was a 67-year-old Swiss man, married and father of two adult sons, living in Geneva with his wife. He used to work as a technical assistant at a government administration service, and he was retired but continued to be very active before his hospitalization. The patient had some risk factors for a severe form of COVID-19 because of his age (greater than 65 years), grade I obesity [body mass index (BMI) of 30.6 kg/m^2^ at his admission], poorly controlled type 2 diabetes, cardiovascular history with arterial hypertension, and ischemic heart disease treated by angioplasty and stent. His usual treatment consisted of acetylsalicylic acid, atorvastatin, olmesartan + hydrochlorothiazide, dapagliflozin + metformin, liraglutide, pantoprazole, and allopurinol. Regarding his psychiatric history, the patient declared having presented a single depressive episode more than 10 years prior with a positive outcome after an unknown antidepressant treatment and follow-up by his general practitioner. He had never been hospitalized in psychiatry and had never had psychiatric follow-up, but according to his wife, he presented periods of hyperactivity, joyfulness, and decreased sleep alternating with periods of depressed mood, social withdrawal, and clinophilia. We understood by questioning the patient that he had never experienced a traumatic event according to the DSM-5: he had never had an experience that put him or someone close to him at risk of serious harm or death or developed posttraumatic stress disorder (PTSD) [8]. Regarding his family history, the patient’s father had suffered from a Pick’s disease (probably type C according to the descriptions) in the context of which he would have presented important psychiatric symptoms (behavior disorders with disinhibition, agitation, etc.).

### Symptoms of the patient and clinical findings

During his first psychiatric evaluation 4 days post-intubation, the patient presented a complex clinical picture. On the first examination, we observed some false memories and amnesia of the period spent in ICU. The patient was convinced that he was in Africa searching for his brother who was SARS-CoV-2 positive and also hospitalized in the same ICU. It is worth mentioning that the patient’s brother had been hospitalized a week before him for SARS-CoV-2, thus our patient also expressed excessive feelings of guilt for probably having transmitted the virus to his brother, with the fear that he could die. He thought that he had been repatriated by helicopter from Africa, while in reality he had been driven by ambulance from his home to the emergency service because of asthenia and fever that had been ongoing for several days. He believed that he had almost died by drowning and that somebody had put a tube in his mouth to kill him. Furthermore, he was suspicious of some caregivers, convinced that he was maltreated and abused while interpreting some of his symptoms (pressure ulcers) in a delusional persecutive mode. He also suffered from false recognitions, believing that he had seen his wife working as a cleaner in the hospital and that she had taken on the post to be by his side. At first, he was convinced the delusions were true but then gradually started to question them. He was oriented in time and space and his speech was coherent. He had no more disturbance of his attention or his consciousness, nor a fluctuation of symptoms, despite the fact that in his medical file he initially presented these symptoms the 4 first days post-intubation, which fulfilled the criteria for delirium. Three main hypotheses for the etiology of the delirium were evoked in this context: (1) PICS could be suspected, because of the physical symptoms (swallowing disorders, hypophonia), cognitive symptoms (amnesia, attention deficit, working memory impairment), and psychiatric symptoms (delusional symptoms, anxiety) following intensive care; (2) SARS-CoV-2-related encephalopathy because of the same symptoms through a mechanism of neurotropism of SARS-CoV-2; (3) acute renal failure. Follow-up by the liaison psychiatry consultation began the fourth day after extubation. We evaluated that he experienced a real risk of death while in the ICU, which validates the condition of a traumatic event before evoking psychotraumatic symptoms that we decided to look for. We found that he had intrusive thoughts linked with false traumatic memories of drowning followed by an aggression and that he did not remember the days in ICU. He presented with emotional lability, anxiety, and sadness. We noticed that his false memories were traumatic and characterized by a feeling of suffocation, possibly related to intubation. He felt insecure with the caregivers. He described flashbacks as if he was drowning again. He was very tense when questioned about the traumatic event. He had assault reactions and hypervigilance. He had some grandiose ideas, mostly noncongruent with the situation (that is, he wanted to initiate his own enterprise for at-home hemodialysis and bioproducts after leaving the hospital). His mood was depressed. He cried easily and felt guilty but without any suicidal ideations. There were no manic symptoms such as disinhibition, familiar contact, logorrhea, sleep disturbances, or mood swings. There was also no flight of ideas. There was distractibility. There was neither psychomotor agitation nor psychomotor slow-down.

### Timeline

The patient was hospitalized for severe ARDS due to SARS-CoV-2 with bacterial infection and septic shock. Because of progressive worsening of respiratory symptoms with increased oxygen requirements, he was transferred to an intensive care unit (ICU) on the fifth day, where he was immediately intubated. During his stay in the ICU, he benefited from protective ventilation, three sessions of prolonged prone position, and curarization during the first 48 hours. The patient was sedated with midazolam, and a propofol relay was performed on the fourth day of ICU hospitalization. A ketamine trial failed at day 7 due to poor tolerance. Fentanyl was administered continuously until the 12th day, at which time all sedation was also stopped. The ICU hospitalization was complicated by progressive but severe worsening of acute renal failure, prompting temporary hemodialysis and a pulmonary septic shock with an undetermined infection requiring broad-spectrum antibiotic therapy. The patient was finally extubated after 13 days of ICU hospitalization and transferred the next day to the internal medicine unit. An ICU diary was written daily during ICU hospitalization by medical students, as with all intensive care patients hospitalized for severe ARDS on SARS-CoV-2. During the first days post extubation, he presented swallowing disorders and hypophonia. He also presented significant emotionality, anxiety, and sadness associated with false memories with delusional content. Thus, a psychiatric evaluation was requested.

### Diagnostic assessment

We referred to the criteria of the Diagnostic and Statistical Manual of Mental Disorders (DSM-5) [8] to investigate the psychiatric diagnosis. In the beginning of the follow-up by the psychiatry liaison, and according to DSM-5, the symptomatology of the patient continued to partially fill the criteria of delirium, with quick disappearance of the disturbance in consciousness and attention (criterion A), fluctuation of symptoms (criterion B), and disturbance in cognition (criterion C). The patient also developed several symptoms of acute stress disorder, according to DSM-5 criteria [8], if we consider that the traumatic event was intensive care for ARDS and the intubation reinterpreted like drowning and associated with fear of death (criterion A). We found ten symptoms of criterion B (nine being needed for diagnosis of ASD): intrusion symptoms with recurrent, involuntary, and intrusive distressing memories of the traumatic event of his drowning (1); dissociative reactions with flashbacks (2); dissociative amnesia (3); intense and prolonged psychological distress when speaking about the traumatic event (4); negative mood (5); dissociative symptoms with an inability to remember an important aspect of the traumatic events partially caused by dissociative amnesia and, in other part, by chemical sedation in ICU (6); arousal symptoms with irritable behavior (7); hypervigilance (8); problems with concentration (9); exaggerated startle response (10) (criterion B). The duration of the disturbance (symptoms in criterion B) was 21 days, extending from 3 days and 1 month after trauma exposure (criterion C). The disturbance caused clinically significant distress in this patient (criterion D), and the disturbance was not attributable to the physiological effects of a substance (criterion E): although the patient had received many types of drugs with risks of psychotropic adverse effects (sedative agents, antibiotics, and corticoids during the treatment of the ARDS), it could not be explained by these as it continued after they were stopped. Given the history of the depressive episode and the affective lability reported, our differential diagnosis also included an eventual manic episode with mixed features (emotionality, emotional lability, excessive guilt, digressive speech, grandiose ideas). Nevertheless, he did not present other basic maniac symptoms (that is psychomotor acceleration, insomnia, and irritability). Thus, the aforementioned diagnostic was ruled out. In the context of a systematic screening test among patients hospitalized in the University Hospital of Geneva (HUG) due to SARS-CoV-2, patients were systematically evaluated on the following scales during the sanitary crisis to promote early management of psychological manifestations related to COVID-19 and a preventive approach: Peritraumatic Dissociative Experiences Questionnaire to assess peritraumatic dissociation (PDEQ) [9], PTSD Check List to assess PTSD (PCL-5) [10], and Hospital Anxiety and Depression Scale (HADS), which helps to discriminate psychic from physical symptoms in patients who suffer from physical symptoms [11]. The patient’s scores at four days post-intubation weer 39 for PDEQ (cutoff > 15), 14 for PCL-5 (cutoff > 32), 5 for HADS-Anxiety (cutoff > 11), and 0 for HADS-Depression (cutoff > 11). The peritraumatic dissociation assessed by the high score on the PDEQ scale indicated an important risk of PTSD [9, 12].

### Therapeutic interventions

Little information and few guidelines are currently available in medical literature for the prevention of PTSD in ICU survivors in the context of COVID-19. Given the high risk of PTSD detected by a history of depressive episode, ASD [6], peritraumatic dissociation as assessed by the high PDEQ score [12], long duration of intubation and sedation (13 days of intubation and 12 days of sedation for this patient) [4], and false memories and amnesia of his intensive care [13], we looked for strategies to prevent progression from ASD to PTSD. We proceeded to the following therapeutic interventions: intensive psychiatric follow-up (three times per week), use of the ICU diary written by the intensive care team, and low-dose antipsychotic treatment with quetiapine 50 mg twice daily with an anxiolytic goal. The aim of our psychotherapeutic approach, which was successful, was to allow him to increase his feeling of security and to cope with the reality of his traumatic experience to reconstruct a coherent and appropriate account of what he experienced when he was in the ICU.

### Follow-up and outcomes

In the course of 3 weeks after the onset of the care by the psychiatry liaison consultation, we noticed a significant improvement of psychiatric symptoms with complete resolution of the acute stress disorder. On the other hand, there were no further signs of delirium. The patient remembered his delusional thoughts and made a clear distinction between his symptoms and reality. He was astonished by his conviction concerning the delusional ideas during the episode and of the proportion of emotional reaction to these ideas. He retained the memory of suffering from chocking but no longer by drowning as in the beginning, but realized it was instead because of SARS induced by SARS-CoV-2. He was helped by reading his ICU diary, which was written daily by medical students and provided to him after his release from intensive care. He understood the successive steps of his care in ICU. That helped him to understand the duration and nature of the different steps of his medical care: 13 days of intubation and then post-intubation chemical sedation, which was slowly decreased from the 11th day, information about his renal failure and hemodialysis, the slow improvement of his attention and consciousness, the nursing care for his toilet needs, and the medical information given to his wife. He came to understand his medical situation and the medical and nursing care that helped him. In view of the favorable evolution of the physical and psychiatric condition of the patient, he was transferred to the rehabilitation unit with a deescalation of psychiatric interventions. He benefited there from a neuropsychological assessment 7 weeks after his admission, which revealed some nonspecific light to moderate attentional deficits and an alteration of working memory. The intensive psychiatric interviews and quetiapine treatment were progressively stopped after full remission of symptoms. The final psychiatric evaluation was 3 months after the beginning of the follow-up, when he was stable and psychiatrically asymptomatic. Three months after the beginning of his follow-up, he again filled out the questionnaires for HADS anxiety and depression and PCL-5 and received the following scores: HADS-Anxiety: 3, HADS-Depression: 1; PCL-5: 9.

## Discussion

There are insufficient details in literature and no official guidelines available for the treatment of psychiatric symptoms in ICU survivors in the context of COVID-19 and the prevention of PTSD. Despite initial very severe psychiatric symptoms, and a high risk of PTSD, this patient had a very favorable and quick clinical improvement that was confirmed at 3 months. He benefited from the psychiatry liaison consultation and the ICU diary written by the intensive care team. This evolution was verified through good evaluation conditions: follow-up for 3 months, allowing a perspective on the patient’s progress, a first early evaluation 4 days after extubation, and the objectification of the psychic state as neurocognitive by the repeated use of validated psychometric tests. This case report illustrates the complexity of the still understudied psychiatric manifestations associated with severe forms of COVID-19. We could have considered that this patient had only a delirium without considering traumatic aspects, although it seems to have been important and useful for the patient to have his traumatic experience recognized. This case highlights that early psychiatric intervention in ICU survivors for COVID-19 reduces the risk of long-term psychiatric issues linked to traumatic effects of COVID-19.

The limitation of the management of this patient is not having proposed a more specific trauma intervention, such as a protocol on recent event of eye movement desensitization and reprocessing (EMDR), to prevent an evolution in the future to PTSD [14] and not to have investigated more protective factors for PTSD and the posttraumatic growth component, which could also help to explain this good evolution. Some recently published data showed encephalopathy manifesting with confusion and delusion in the early stage of SARS-CoV-2 infection [1], presumably due to the neurotropism of SARS-CoV-2 [15]. Other data indicate a significant traumatic effect of the experience of SARS-CoV-2 infection, with evolution to PTSD in 28% of adults surviving COVID-19 [7]. The case of this patient shows the complexity of the psychiatric effects of COVID-19 because of the entanglement of physical symptoms due to immunoinflammatory mechanisms and psychiatric symptoms due to stress. This patient was objectively exposed to a death threat experience in the context of his hospitalization in the ICU for a severe form of COVID-19, a circumstance that qualifies as a traumatic event. After extubation, he suffered from delirium with false memories. Traumatic memory has been well investigated, and some mechanisms of dissociation can explain peritraumatic amnesia but also some false memories [14]. The dissociation symptoms could underlie the impaired encoding of traumatic memory [16], which increases the risk of PTSD [12]. The mechanisms that can be involved in false memories for this patient can be both the chemical sedation and the dissociation, both of which are known to increase the risk of post-ICU PTSD [4, 5].

The patient was reevaluated by psychiatric interview at 3 months after the episode. He noted that it was a very intense experience that changed his point of view on life in a positive way, suggesting a posttraumatic growth component. He expressed his gratitude for the care provided by the psychiatry liaison consultation team.

## Conclusion

The COVID-19 pandemic has had a serious impact on global mental health, especially in ICU survivors. There are insufficient details in literature and no official guidelines available for the treatment and follow-up of ASD and prevention of PTSD in the context of COVID-19. This case report illustrates how a delusional clinical presentation after intensive care can hide psychotraumatic symptoms that should be systematically looked for after intensive care. The systematic screening for symptoms of peritraumatic dissociation for this patient helped us to identify the risk of PTSD. Early intervention by psychiatry liaison consultation with intensive psychiatric follow-up, use of the ICU diary written by the intensive care team, and low doses of antipsychotic prevented development of PTSD at 3 months in this patient. Further studies are needed on the psychiatric effects of COVID-19 and how to avoid PTSD in ICU survivors of SARS-CoV-2.

## Data Availability

Not applicable.

## Abbreviations

ARDS: Acute respiratory distress syndrome
ASD: Acute stress disorder
BMI: Body mass index
DSM5: Diagnostic and Statistical Manual of Mental Disorders
HADS: Hospital Anxiety and Depression Scale
HUG: University Hospital of Geneva
ICU: Intensive care unit
PCL-5: PTSD Check List
PDEQ: Peritraumatic Dissociative Experiences Questionnaire
PICS: Post intensive care syndrome
PTSD: Posttraumatic stress disorder
